# Supplementary material for: Facilitators and barriers for healthcare workers’ adherence to the national nutritional guidelines for people living with HIV in Dar-es-Salaam: A mixed-method study
Source: PLOS Glob Public Health. 2025 Feb 25;5(2):e0003664. doi: 10.1371/journal.pgph.0003664 (PMC11856515; doi:10.1371/journal.pgph.0003664)
Supplement: S1 Text — (PDF) [file pgph.0003664.s001.pdf]

**OBSERVATION CHECKLIST ADOPTED FROM USAID & FANTA NACS evaluation tool.**

**Questionnaire adopted from TDHS and THIS.**

SECTION A.

| |
|---|
| THE OBSERVATION OF THE PROVIDER-CLIENT INTERACTION. |
| THIS TOOL IS USED TO CONDUCT A SPECIFIED NUMBER OF OBSERVATIONS IN EACH FACILITY. CONSENT MUST BE OBTAINED FROM THE PROVIDER AND THE CLIENT. |
| FACILITY INFORMATION |
| 01 NAME OF FACILITY____ FACILITY CODE ........................................................<br>02 NAME OF UNIT UNIT CODE .................................................................<br>03 DISTRICT______ DISTRICT CODE ......................................................<br>04 TYPE OF FACILITY_____ TYPE OF FACILITY CODE ...................................... |
| PROVIDER INFORMATION |
| 06 PROVIDER TYPE CODE<br>.................................................................................................................................................... ☐☐<br>07 SEX OF PROVIDER (1=MALE; 2=FEMALE)<br>............................................................................................................. |
| OBSERVATION INFORMATION |
| 08 DATE ........................................................................................ Day Month Year<br>09 OBSERVER NAME_____ OBSERVER CODE .................................... ☐☐ |

Provider Code.

PEDIATRICIAN 1

OB/GYN 2

GENERAL PHYSICIAN 3

MEDICAL OFFICER 4

NURSE 5

MIDWIFE 6

NURSING ASSISTANT, AID, AUXILLIARY 7

NUTRITIONIST 8

HEALTH EDUCATOR / SOCIAL WORKER / COUNSELOR 9

COMMUNITY HEALTH AGENT 10

| CHECKLIST FOR OBSERVATION. | | | | | | | |
|---|---|---|---|---|---|---|---|
| OBSERVE THE PROVIDER'S ACTIONS AND INTERACTIONS WITH THE CLIENT. EACH OBSERVATION POINT, MARK:<br>• 0 (NO), THE PROVIDER DID NOT PERFORM THE ACTION;<br>• 1 (YES), YOU OBSERVED IT AND IT WAS DONE POORLY, INCORRECTLY, OR ONLY PARTIALLY;<br>• 2 (YES), YOU OBSERVED IT AND IT WAS DONE WELL AND COMPLETELY;<br>• 8 FOR "DON'T KNOW" IF YOU WERE UNABLE TO OBSERVE THE ENTIRE ACTION; OR<br>• 9 IF IT IS "NOT APPLICABLE (NA)". NOTE THAT THIS OPTIONAL RESPONSE SHOULD ONLY BE USED WHERE THE CODE IS PROVIDED AND THE CELL IS <u>NOT</u> SHADED. | | | | | | | |
| NO. | ACTION | YES, DONE WELL (2) | YES, DONE POORLY, INCORRECTLY, OR PARTIALLY (1) | NOT DONE (0) | DK (8) | NA (9) | REMARKS |
| ASSESSMENT | | | | | | | |
| 01. | RECORD SEX OF THE CLIENT HERE. | MALE 1<br>FEMALE 2 | | | | | |
| CLIENT'S WELL BEING | | | | | | | |
| 02. | Did the healthcare provider inquire about the patient's reason for visiting? | 2 | 1 | 0 | 8 | | |
| 03. | NOTE THE PURPOSE. AFFECTION: AFTER THE CONSULTATION, REVIEW THE CLIENT CHART OR THE PROVIDER'S NOTES. | ______________________<br>______________________ | | | | | |
| 04. | Did the healthcare provider verify the client's age or check the age that was noted on another document in the facility? | 2 | 1 | 0 | 8 | | |
| 05. | INSERT THE CLIENT'S BIRTH DATE HERE. EXAMINING THE CLIENT CHART OR THE PROVIDER'S NOTES | DAY ................................................................. .. | | | | | |

| | | | |
|---|---|---|---|
| | FOLLOWING THE CONSULTATION MAY BE NECESSARY FOR THIS. | MONTH ................................................................<br><br>YEAR ..........................................................<br><br>NOT AVAILABLE 999 | |
| 06. | RECORD THE CLIENT'S AGE HERE. IF NEEDED, REVIEW THE CLIENT CHART OR THE PROVIDER'S NOTES AFTER THE CONSULTATION. CIRCLE THE CODE INDICATING IF THE AGE IS RECORDED IN MONTHS OR YEARS, THEN RECORD AGE IN THE SPACE PROVIDED. CIRCLE '999' IF<br><br>THE AGE IS NOT AVAILABLE. | A. AGE IN MONTHS 1<br>B. AGE IN YEARS 2<br>NOT AVAILABLE 999 | |

| | | | | | | | |
|---|---|---|---|---|---|---|---|
| 07. | Did the healthcare provider weigh the patient or check the weight recorded elsewhere in the building? | 2 | 1 | 0 | 8 | | |

| | | | |
|---|---|---|---|
| 08. | NOTE THE WEIGHT AND UNIT OF MEASUREMENT (E.G., METERS, CENTIMETERS) OF THE CLIENT HERE. EXAMINING THE CLIENT CHART OR THE PROVIDER'S NOTES FOLLOWING THE CONSULTATION MAY BE NECESSARY FOR THIS. | WEIGHT .................................................... .<br><br>UNIT OF MEASURE ________<br><br>NOT AVAILABLE 999 | |

| | | | | | | | |
|---|---|---|---|---|---|---|---|
| 09. | Did the healthcare worker take the client's height and/or length measurements, or did they check them elsewhere in the building? | 2 | 1 | 0 | 8 | | |

| | | | |
|---|---|---|---|
| 10. | INSERT THE CLIENT'S HEIGHT AND UNIT OF MEASUREMENT (E.G., POUNDS, KILOGRAMS) HERE. AFTER THE CONSULTATION, REVIEW THE CLIENT CHART OR THE PROVIDER'S NOTES, IF NEEDED. | HEIGHT ..................................................... .<br><br>UNIT OF MEASURE ________<br><br>NOT AVAILABLE 999 | |

| | | | | | | | |
|---|---|---|---|---|---|---|---|
| 11. | Did the healthcare worker assess the client's MUAC or go over the MUAC measured somewhere else in the building? | 2 | 1 | 0 | 8 | | |
| 12. | MUAC AND UNIT OF MEASUREMENT (E.G., CENTIMETERES) OF THE CLIENT SHOULD BE NOTED HERE. AFTER THE CONSULTATION, REVIEW THE CLIENT CHART OR PROVIDER'S NOTES, IF NEEDED. | MUAC.......................................... [ ][ ][ ] . ................<br>UNIT OF MEASURE _______<br>NOT AVAILABLE 999 | | | | | |
| 13. | Did the health worker categorize the nutritional status or evaluate a previous classification made in the facility? | 2 | 1 | 0 | 8 | | |
| 14. | MARK THE CODE<br>CORRESPONDING WITH THE PROVIDER'S CLASSIFICATION OF<br>THE CLIENT'S NUTRITIONAL<br>STATUS. IF NEEDED, REVIEW THE CLIENT CHART OR THE PROVIDER'S NOTES AFTER THE CONSULTATION. | SEVERELY MALNOURISHED 1<br>MODERATELY MALNOURISHED 2<br>NORMAL 3<br>OVERWEIGHT 4<br>OBESE 5<br>OTHER 6<br>SPECIFY: _____<br>NOT CLASSIFIED 9 | | | | | |
| 15. | Did the health care worker examine the client's results from screenings conducted elsewhere in the facility or check the client for anemia (pale palms or inner eyelids)? | 2 | 1 | 0 | 8 | | |
| 16. | Did the healthcare worker give or distribute any advice regarding nutrition, including how to feed or eat? | 2 | 1 | 0 | 8 | | |

| | | | | | | | |
|---|---|---|---|---|---|---|---|
| 17. | Did the healthcare worker recommend community-based nutrition support services to the client? | 2 | 1 | 0 | 8 | | |
| 18 | Did the healthcare provider refer a community-based health care provider to the client?? | 2 | 1 | 0 | 8 | | |

SECTION B.

| Patient demographics and disease specific. | | |
|---|---|---|
| 1. | Patient sex | a) Female<br>b) Male |
| 2. | In what month and year where you born? | Month . . . . . . . . .. .<br>Don't Know Month. ........98<br>Year . . . . . . . . . . . . . . .<br>Don't Know Year ....... . 9998 |
| 3. | Where do you live? | Urban................1<br>Rural..............2 |
| 4. | Have you ever attended school? | Yes . . . . . . . . . . . . . . . . . . . .1<br>No . . . . . . . . . . . . . . . . . . . . . 2 |
| 5. | What is the highest level of school you attended:<br>Primary, secondary, or higher? | Primary . . . . . . . . . . . . . 1<br>Secondary . . . . . . . . . . .2<br>Higher . . . . . . . . . . . . . . . 3 |
| 6. | Have you done any work in the last 12 months for which you received cash or goods as payment? | Yes = 1<br>No = 2<br>Don’t Know = 8<br>Refused = 9 |

| | | |
|---|---|---|
| 7. | Have you done any work in the last seven days for which you received cash or goods as payment? | Yes = 1<br>No = 2<br>Don't Know = 8<br>Refused = 9 |
| 8. | What is your marital status now: are you widowed, divorced, or separated? | Never Married....................1<br>Married............................2<br>Living together. . . . . . . . . . . . . . . ... 3<br>Divorced/separated . . . . . . . . . . . . . . . .  4<br>Widowed..............5 |
| 9. | What month and year did (NAME) last see a doctor, clinical officer or nurse for HIV medical care | Month . . . . . . . . . .. .<br>Don't Know Month. ........98<br>Year . . . . . . . . . . . . . . .<br>Don't Know Year ....... . 9998 |
| 10. | Has (NAME) ever had a CD4 count test? The CD4 count tells you how sick you are with HIV and if you need to take ARVs or other HIV medications | Yes = 1<br>No = 2<br>Don't Know = 8<br>Refused =9 |
| 11. | What month and year was (NAME) last tested for his/her CD4 count? | Month . . . . . . . . . .. .<br>Don't Know Month. ........98<br>Year . . . . . . . . . . . . . . .<br>Don't Know Year ....... . 9998<br>Count .......... |

SECTION C.

| Adherence Level; based on TFNC recommendation and Index Measuring Scoring | | |
|---|---|---|
| NO | QUESTION | RESPONSE |
| 1. | Did the healthcare worker conduct a clinical assessment? | Yes = 1<br>No = 0<br>Don't Know = 8 |

| | | |
|---|---|---|
| 2. | Did the healthcare worker conduct anthropometric assessment? | Yes = 1<br>No = 0<br>Don't Know = 8 |
| 3. | Did the healthcare worker conduct biochemical assessment? | Yes = 1<br>No = 0<br>Don't Know = 8 |
| 4. | Did the healthcare worker conduct a dietary assessment? | Yes = 1<br>No = 0<br>Don't Know = 8 |
| 5. | Did the healthcare worker explain to you about the side effects of medications and dietary management? | Yes = 1<br>No = 0<br>Don't Know = 8 |
| 6. | Did the healthcare worker advise you on food and water safety and hygiene? | Yes = 1<br>No = 0<br>Don't Know = 8 |
| 7. | Did the healthcare worker give counsel about nutrition and HIV? | Yes = 1<br>No = 0<br>Don't Know = 8 |
| 8. | Did the healthcare worker give you any micronutrient supplements? | Yes = 1<br>No = 0<br>Don't Know = 8 |
| 9. | Did the healthcare worker scheduled you for follow up care? | Yes = 1<br>No = 0<br>Don't Know = 8 |

**Health Facility Unit In charge/Manager.**

**Questionnaire adopted from USAID & FANTA rapid evaluation tool on NACS.**

| FACILITY INFORMATION |
|---|
| 01 NAME OF FACILITY____ FACILITY CODE.............................<br>02 PATIENT VOLUME______________________<br>03 DISTRICT_____ DISTRICT CODE............................<br>04 TYPE OF FACILITY____ TYPE OF FACILITY CODE.............. |
| INTERVIEW INFORMATION |
| 05 DATE.................................................................Day Month Year<br>06 INTERVIEWER NAME__ INTERVIEWER CODE........................... |

| EQUIPMENT AND TOOLS FOR ASSESSMENT OF NUTRITION STATUS | | | |
|---|---|---|---|
| Now I would like to ask you questions about the equipment and tools available in your facility for providing nutrition services. | | | |
| 01. | Is an adult weighing scale available in your facility?<br>IF YES, ASK: Is it assigned to this facility or shared with another facility? | YES, ASSIGNED 1<br>YES, SHARED 2<br>NO 3<br>N/A 9 | <br><br>111_1<br>111_1 |
| 02. | How many are in working condition?<br>RECORD THE NUMBER IN THE SPACE PROVIDED. FILL IN LEADING ZEROES AS NECESSARY. RECORD ‘88’ IF NOT KNOWN. | | |
| 03. | RECORD THE DAY, MONTH, AND YEAR OF THE LAST CALIBRATION IF UNKNOWN, RECORD ‘88’ OR ‘8888’ IN THE BOXES PROVIDED. | DAY............................<br>MONTH....................<br>**YEAR............** | |

| | | | |
|---|---|---|---|
| 04. | Is a stadiometer (for adults) available in your facility?<br><br>IF YES, ASK: Is it assigned to this facility or shared with another facility? | YES, ASSIGNED 1<br>YES, SHARED 2<br>NO 3<br>N/A 9 | <br><br>□ 113_1<br>□ 113_1 |
| 05. | How many are in working condition??<br><br>RECORD THE NUMBER IN THE SPACE PROVIDED. FILL IN LEADING ZEROES AS NECESSARY. RECORD '88' IF NOT KNOWN. | [ ][ ] | |
| 06. | Is a MUAC tape for adults available in your facility?<br><br>IF YES, ASK: Is it assigned to this facility or shared with another facility? | YES, ASSIGNED 1<br>YES, SHARED 2<br>NO 3<br>N/A 8 | <br><br>□ 115_1<br>□ 115_1 |
| 07. | How many are available?<br><br>RECORD THE NUMBER IN THE SPACE PROVIDED. FILL IN LEADING ZEROES AS NECESSARY. RECORD '888' IF NOT KNOWN. | | |

| HUMAN RESOURCES | |
|---|---|
| Now I am interested in asking you questions about human resources and human resource management. | |
| | First, could you tell me how many of each kind and cadre of health workers are employed by this facility? The only medical professionals that interest me are those that work for this particular hospital. Volunteers and workers on secondment are not included in this.<br><br>A. READ THE PROVIDER CADRE, THEN ASK: How many PROVIDERCADRE are allocated to the [UNIT NAME] unit?<br><br>IN THE ALLOCATED SPACE, NOTE THE NUMBER OF PROVIDERS. If the respondent is unsure, note "99."<br><br>B. NEXT ASK: Does [PROVIDER TYPE] usually provide nutrition services in this facility? EXPLAIN: By nutrition services, I mean assessment of nutritional status, nutrition counseling, nutrition support, and/or referral to nutrition support services.<br><br>NOTE HOW MANY PROVIDERS ARE USUALLY AVAILABLE IN THE ALLOCATED SPACE TO PROVIDE NUTRITION SERVICES FOR? Make a note of "99" if the respondent is unsure.<br><br>C. FINALLY, ASK: Has [PROVIDER TYPE] been trained to provide nutrition services in this facility? EXPLAIN: By training, I mean pre-service or in-service training. |

| | |
|---|---|
| | RECORD THE NUMBER OF PROVIDERS THAT HAVE BEEN TRAINED IN PROVIDING NUTRITION SERVICES IN THE SPACE PROVIDED. IF THE RESPONDENT DOES NOT KNOW, RECORD ‘99’.<br><br>Note: Revise provider types/cadres and units as well as the type of training as appropriate for the country context and evaluation objectives. |

| **Provider Type / Cadre** | | **A. How many [PROVIDER TYPE] are assigned to the [UNIT NAME] unit?** | | | **B. How many [PROVIDER TYPE] usually provide nutrition services in the [UNIT NAME] unit?** | | | **C. How many [PROVIDER TYPE] have been trained in the past three years to provide nutrition services in the [UNIT NAME] unit?** | | |
|---|---|---|---|---|---|---|---|---|---|---|
| | | **[UNIT NAME]** | | | **[UNIT NAME]** | | | **[UNIT NAME]** | | |
| | | (a) ANC | (c) PEDIATRIC | (d) HIV/TB | (a) ANC | (c) PEDIATRIC | (d) HIV/TB | (a) ANC | (c) PEDIATRIC | (d) HIV/TB |
| 125_1 | PEDIATRICIAN | | | | | | | | | |
| 125_2 | OB/GYN | | | | | | | | | |
| 125_3 | GENERAL PHYSICIAN | | | | | | | | | |
| 125_4 | MEDICAL OFFICER | | | | | | | | | |
| 125_5 | NURSE | | | | | | | | | |
| 125_6 | MIDWIFE | | | | | | | | | |
| 125_7 | NURSING ASSISTANT, AID, AUXILLIARY | | | | | | | | | |
| 125_8 | NUTRITIONIST | | | | | | | | | |
| 125_9 | HEALTH EDUCATOR / SOCIAL WORKER / COUNSELOR | | | | | | | | | |

| MENTORING / COACHING/TRAINING | | | |
|---|---|---|---|
| Now I would like to ask you about mentoring/coaching/training conducted in this facility. | | | |
| 01. | Are healthcare staff members at this medical facility ever supervised, coached, or trained in providing nutrition services? | YES 1<br>NO 2<br>DON'T KNOW 8 | |
| | IF YES, ASK: Could you explain the training, coaching, and mentorship that you received? IF NOT, QUESTION WHY NOT?<br>__________<br>__________<br>__________ | | |

| SUPERVISION & FEEDBACK | | | |
|---|---|---|---|
| Next, I would like to ask you about any supervision of health providers that is conducted in this facility. | | | |
| 01. | Do those working with nutrition services in this facility ever have supervision? | YES 1<br>NO 2<br>DON'T KNOW 8 | <br>□130<br>□130 |
| | IF YES, ASK: Could you describe the supervisory visits? IF NO, ASK: Why not?<br>__________<br>__________<br>__________ | | |
| 02. | How many times in the past year have nutrition service providers in this facility been supervised? | NEVER 0<br>ONCE 1<br>2-3 TIMES 2<br>4-5 TIMES 3<br>≥ 6 TIMES 4<br>DON'T KNOW 8 | |
| 03. | Are nutrition service providers given feedback—positive or negative—based on their supervision? | YES 1<br>NO 2<br>DON'T KNOW 8 | |

**Interview guide: Healthcare worker.**

INTERVIEW NO: ..........................

STARTING TIME: ..........................

HEALTHCARE PROVIDER PERSONAL INFORMATION.

1. Current Age.................
2. Gender ..............
3. What is your profession.......................
4. How many years have you worked in the CTC clinic?............................

*Facilitators*

- Regular training programs:

How often do you get access to regular training programs on the National Guideline for Nutritional Care and Support of People Living with HIV? what do you think is lacking for your competencies? How helpful have these training programs been in helping you to adhere to the guidelines? probe

- Supportive leadership:

Does your leadership team promote a culture of adherence to the guidelines? What are some specific ways that your leadership team supports you in implementing the guidelines?

- Integration of nutritional care:

How is nutritional care integrated into other healthcare services at your clinic? How does this integration help you to provide more comprehensive care to your patients?

- Peer support networks:

Are there any peer support networks available to people living with HIV in your area? How can these networks be leveraged to support adherence to the guidelines?

- Patient education initiatives:

What patient education initiatives are in place at your clinic to help people living with HIV understand the importance of good nutrition and how to follow the guidelines? How effective have these initiatives been?

- Availability of resources:

Do you have access to the resources (e.g., staff, time, funding, equipment, supplies) you need to effectively implement the guidelines? How do any resource limitations impact your ability to adhere to the guidelines?

*Challenges*

- Staff turnover:

Can you describe any challenges you have faced with staff turnover? What impact has this had on your ability to adhere to the guidelines?

- Lack of funding for nutritional assistance:

Can you describe any challenges you have faced with lack of funding for nutritional assistance? What impact has this had on your ability to adhere to the guidelines?

- Work environment barriers:

Can you describe any work environment barriers that have hindered your ability to effectively implement the guidelines? (e.g., high patient load, time constraints, poor infrastructure, shortage of supplies, heavy workload)

- Inconsistent policy implementation:

Can you describe any challenges you have faced with inconsistent policy implementation across CTCs? What impact has this had on your ability to adhere to the guidelines?

Interviewee's Opinions.

- What are your recommendations for improving the implementation of the National Guideline for Nutritional Care and Support of People Living with HIV in CTC clinics?
- What are your hopes for the future of nutrition care for people with HIV in Tanzania?
- Are there any other challenges that have hindered your ability to adhere to the guidelines?
