# Supplementary material for: Facilitators and barriers for healthcare workers’ adherence to the national nutritional guidelines for people living with HIV in Dar-es-Salaam: A mixed-method study
Source: PLOS Glob Public Health. 2025 Feb 25;5(2):e0003664. doi: 10.1371/journal.pgph.0003664 (PMC11856515; doi:10.1371/journal.pgph.0003664)
Supplement: Attachment — Submitted filename: Response to Reviewers.docx [file pgph.0003664.s005.docx]

**COMMENTS MATRIX.**

Reviewer comments and Authors remarks on the article titled: **Facilitators and barriers for healthcare workers' adherence to the national nutritional guidelines for people living with HIV in Dar-es-Salaam: A mixed-method study;**

| **SECTION** | **REVIEWER COMMENTS** | **AUTHORS REMARKS** |
| --- | --- | --- |
| Title and abstract | Title is clear and addressed an important issue of health significance  Abstract is unstructured and presented the findings within reasonable accepted word limits (224 words). However, authors need to mention the gap for the study and the study period. | Thank you for your valuable feedback and for recognizing the significance of our study.  We have revised the manuscript and structured the abstract We have mentioned the gap (Line 4-6) and ensured the ensured the word limit is not exceeded. |
| Introduction, Background/rationale | The scientific background and rationale are well addressed though the language needs some improvement. | Thank you for your constructive comment. We have edited the entire manuscript to ensure clarity and improved language. |
| Objectives | Clear |  |
| Methods | The design, settings, and participants are well described. The eligibility criterion needs to be properly addressed.  Data collection and analysis are described though still some improvements are needed (who were the data collectors? Any training given? how was anthropometric measurements were taken, data quality issues need to be mentioned, etc,).  The Statistical methods employed are sufficient. | Thank you for your thoughtful feedback.  We have addressed the eligibility criteria (Line 80-86),  We have revised the data collection section and clarify the data collectors and training they received. (Line 138).  We further edited the section on data collection and explain how anthropometric assessment was done including data quality issues Line (112-125). |
| Results | Except for some minor language, otherwise the findings are described in 4 tables and 2 figures.  Line 185 needs to be corrected.  The Tables need some more descriptions (Table 1). | Thank you for insightful feedback, we have corrected the Table and added more description on the respective table Line (227-230). |
| Discussion | The key findings are summarized with reference to study objectives though the language is not clear. | Thank you for your constructive feedback. We have revised the manuscript to improve the language and ensure that the findings were articulated more clearly (Line 297-354) |
| Conclusion: | Based on the findings and highlighted the action points. | Thank you for your constructive feedback. We have revised the conclusion to highlight the action points. (Line 356-414) |
| References | Adequate but not consistent in terms of the style. | Thank you for your feedback. We have reviewed and revised the references that were inconsistent (Line 424-527) |

| **Section** | **Reviewer 1** | **Author’s Remarks** |
| --- | --- | --- |
| Methods. | Authors need to improve the methods section which lacks the most important elements such as how anthropometric measurements were taken; mention who collected the data; was training given to data collectors and explicitly mention how data quality was assured. Secondly some improvements in the language is also necessary | Thank you for your constructive feedback and the opportunity to improve our manuscript. We have revised the methods section to include details, data collectors' qualifications and training (Line 131-135) and data quality assurance measures (Line138-139). We also improved the language for clarity.  The anthropometric assessment involved observing whether healthcare providers conducted the necessary measurements, such as height, weight and BMI, for PLHIV. (Line 112-125). |

| **Section** | **Comments Reviewer 2** | **Author’s Remarks** |
| --- | --- | --- |
| Introduction | Line 2: Could you please update the data to reflect HIV statistics for 2023, rather than 2022, in the second line? | Thank you for your insightful feedback. We have revised the statistics at (Line 22). |
|  | Line 52: You mention progress towards the 95-95-95 targets. Could you elaborate on the current status of the 95-95-95 target in Tanzania? Please include this information in your introduction, and discuss its implications on malnutrition. | We have now included the requested details regarding the 95-95-95 targets and their implications on malnutrition at the introduction level (Line 44-48). |
| Methods | Lines 63-65: In the "Study Design" section, the statistics provided here can be removed and integrated into the introduction. | We appreciate this insightful feedback; we have made changes in the introduction by adding the statistics in Line 30-33) |
|  | Lines 71-72: Can you provide more details regarding why 7 participants (1.46%) refused to participate in the study? | Thank you for your feedback. We have provided additional details about the seven participants (1.46%) who did not to participate in the study (Line 87-88) |
|  | Regarding your semi-structured questionnaire for qualitative data;  Was it developed specifically for this study, or adopted from previous studies? | The semi-structured qualitative questionnaire was developed specifically for this study based on literature review. |
|  | If it was developed for this study, please include the interview guide as a supplementary file. If it was adopted from previous studies, please provide references. | We have attached the interview guide as a supplementary file. |
|  | Did you follow all the necessary steps for reporting qualitative studies? If so, please submit the Consolidated Criteria for Reporting Qualitative Research (CORE-Q) as supplementary files. | Thank you for your valuable feedback. We followed all necessary steps for reporting qualitative studies and submitted the Consolidated Criteria for Reporting Qualitative Research (CORE-Q) and attached as supplementary file. |
| Findings | Line 181: Is this section presenting quantitative or qualitative results? If it is part of the qualitative findings, could you provide a table detailing your participants' experiences and professional backgrounds? | Thank you for your valuable feedback. We have clarified that this section presented qualitative findings and included a table detailing participants' experiences and professional backgrounds. We believed this enhancement provided a clearer understanding of the data (Line 227-230) |
| Limitation | Please ensure that the study’s potential limitations are clearly highlighted. | We have revised the manuscript to clearly outline the study's limitations, emphasizing the constraints on external validity due to the unique context of Dar es Salaam and the potential for response bias from partially self-reported data (Line 350-354). |
| Conclusion | Finally, the conclusion is too long. Kindly revise it to deliver a clearer, more concise message for the reader. | Thank you for your constructive feedback. We have revised the conclusion to ensure it is clearer and more concise for the reader (Line 356-414). |
