# Supplementary material for: Facilitators and barriers for healthcare workers’ adherence to the national nutritional guidelines for people living with HIV in Dar-es-Salaam: A mixed-method study
Source: PLOS Glob Public Health. 2025 Feb 25;5(2):e0003664. doi: 10.1371/journal.pgph.0003664 (PMC11856515; doi:10.1371/journal.pgph.0003664)
Supplement: Attachment — Submitted filename: plose-HIV-2024.docx [file pgph.0003664.s002.docx]

My reflections on the article titled “Facilitators and barriers for healthcare workers' adherence to the national nutritional guidelines for people living with HIV in Dar-es-Salaam: A mixed-method study;

| Title and abstract |  | Title is clear and addressed an important issue of health significance  Abstract is unstructured and presented the findings within reasonable accepted word limits (224 words). However, authors need to mention the gap for the study and the study period. | |
| --- | --- | --- | --- |
| Introduction, Background/rationale | | The scientific background and rationale is well addressed though the language needs some improvement. | |
| Objectives |  | Clear | |
| Methods |  | The design, settings, and participants are well described.  The eligibility criterion needs to be properly addressed.  Data collection and analysis are described though still some improvements are needed (who were the data collectors? Any training given? how was anthropometric measurements were taken, data quality issues need to be mentioned, etc,) .  The Statistical methods employed are sufficient. | |
| Results |  | Except for some minor language, otherwise the findings are described in 4 tables and 2 figures.  Line 185 needs to be corrected.  The Tables need some more descriptions (Table 1). | |
| Discussion |  | The key findings are summarized with reference to study objectives though the language is not clear. | |
| Conclusion: |  | Based on the findings and highlighted the action points. | |
| References |  | Adequate but not consistent in terms of the style. | |
