# Supplementary material for: Facilitators and barriers for healthcare workers’ adherence to the national nutritional guidelines for people living with HIV in Dar-es-Salaam: A mixed-method study
Source: PLOS Glob Public Health. 2025 Feb 25;5(2):e0003664. doi: 10.1371/journal.pgph.0003664 (PMC11856515; doi:10.1371/journal.pgph.0003664)
Supplement: Attachment — Submitted filename: Feedback for authors- plosone.docx [file pgph.0003664.s004.docx]

**Date: 24-12-16**

**Study Title: Facilitators and barriers for healthcare workers' adherence to the national nutritional guidelines for people living with HIV in Dar-es-Salaam: A mixed-method study**

**Comments: Authors addressed all comments**

**Recommendation : accepted for publication**
